# Long‐Term Ultrasound Surveillance of Solid and Predominantly Solid Thyroid Nodules Reveals Two Distinct Absorption Patterns

**DOI:** 10.1155/rrp/5896301

**Published:** 2026-01-20

**Authors:** Yan Hu, Wei Zhou, Shangyan Xu, Guiping Zhang, Xiaoling Ma, Lu Zhang, Weiwei Zhan

**Affiliations:** ^1^ Department of Ultrasound, Ruijin Hospital, Shanghai Jiaotong University School of Medicine, Shanghai, 200025, China, shsmu.edu.cn; ^2^ Department of Ultrasound, Shanghai Jiao Tong University Medical School Affiliated Ruijin Hospital Luwan Branch, Shanghai, 200020, China

**Keywords:** absorption, diagnostic imaging, follow-up, thyroid nodules, ultrasound

## Abstract

**Background:**

To characterize volume reduction in benign solid and predominantly solid thyroid nodules during long‐term ultrasound surveillance, and to describe distinct absorption patterns over time.

**Methods:**

This retrospective study included 34 solid or predominantly solid thyroid nodules from 32 patients (median age, 46 years; 78.1% female), who underwent longitudinal ultrasound surveillance for a median of 28 months (interquartile range, 47–120 months). Volumetric measurements were obtained at multiple time points. Generalized additive mixed models (GAMMs) were employed to model nonlinear trends in volume over time. Unsupervised clustering was applied to identify representative regression trajectories based on individual volume curves. Clinical and ultrasound features were compared between trajectory‐defined groups.

**Results:**

All nodules exhibited measurable volume decrease over time (median reduction: 85.6%). Two representative volume change patterns were identified: a rapid‐absorption group with early shrinkage and a slow‐absorption group with gradual decline. Nodules in the latter were more frequently located in the lower pole and lacked Doppler flow at baseline (*p* < 0.05). Some nodules developed hypoechoic or stiff appearances over time, leading to higher TIRADS categories despite continuous shrinkage and no clinical signs of malignancy.

**Conclusion:**

Solid and predominantly solid thyroid nodules can undergo significant volume reduction during long‐term surveillance, resembling the well‐documented absorption phenomenon of cystic nodules. This phenomenon may be underrecognized in clinical practice. Describing the diversity of volume change patterns may improve the understanding and interpretation of nodule evolution during follow‐up.

## 1. Introduction

Thyroid nodules are frequently detected in adults, with ultrasonography identifying nodules in up to 25% of individuals undergoing routine imaging [1, 2]. Purely cystic nodules are well recognized to follow a benign course, often regressing or disappearing spontaneously [3]. During this process, ultrasonography typically reveals characteristic features such as cavity collapse, disappearance of anechoic fluid, and internal echogenic debris [4]. The “mummified sign,” representing a shrunken cavity or desiccated contents, is considered a hallmark of benign cystic involution [5–9]. Such nodules are classified as very low risk in systems such as the American Thyroid Association (ATA), the American College of Radiology Thyroid Imaging Reporting and Data System (ACR TIRADS), and the Chinese‐TIRADS (C‐TIRADS) and typically require minimal follow‐up [10–12].

Solid and predominantly solid thyroid nodules are conventionally regarded as structurally stable lesions that rarely show size reduction during follow‐up. These nodules carry a higher malignancy risk on ultrasonography [13–15]. In clinical practice, however, a subset of them demonstrates measurable volume reduction and internal remodeling during surveillance, accompanied by changes in echogenicity, stiffness, or vascularity [3]. Such evolving appearances may resemble malignant transformation and often lead to diagnostic uncertainty, unnecessary fine‐needle aspiration (FNA), or overtreatment.

Most previously reported “absorption” phenomena have involved cystic or cyst‐dominant nodules, in which regression is attributed to gradual fluid resorption [6, 16, 17]. Whether nodules with a predominantly solid composition can exhibit a comparable process has not been systematically investigated. The absence of such data limits understanding of benign nodule evolution and may contribute to misinterpretation of longitudinal ultrasound findings.

The purpose of this study was to systematically characterize volume reduction and sonographic evolution in solid and predominantly solid thyroid nodules during long‐term ultrasound surveillance. Longitudinal volume trajectories were analyzed using generalized additive mixed modeling (GAMM) and unsupervised clustering to identify representative absorption patterns. By delineating the imaging and temporal features associated with these patterns, this study provides a descriptive reference for recognizing benign structural remodeling and improving the interpretation of follow‐up ultrasound examinations.

## 2. Materials and Methods

### 2.1. Study Design and Participants

This retrospective cohort study was approved by the institutional review board of the corresponding institution (Approval No. 2025122), with a waiver of informed consent due to its observational nature. This study was performed following the STROBE guideline (Supporting Information available (here)).

Inclusion criteria were as follows: (1) solid or predominantly solid thyroid nodules on initial ultrasonography, with documented reduction in maximal diameter or volume observed on at least two follow‐up ultrasound examinations; (2) a minimum ultrasound follow‐up duration of more than 12 months; and (3) availability of complete clinical records and serial imaging data. Exclusion criteria included (1) patients younger than 18 years at the time of initial evaluation and (2) nodules with prior interventional therapy (such as chemical ablation or thermal ablation) or surgery.

### 2.2. Ultrasound Evaluation

All examinations were performed with the patients in the supine position and the neck slightly extended to fully expose the thyroid region. Each thyroid lobe and the isthmus were scanned systematically in both transverse and longitudinal planes to cover the entire gland and adjacent cervical soft tissues. All nodules were evaluated using high‐resolution grayscale ultrasonography and color Doppler imaging, with elastography performed when available. Gain, depth, and focus were adjusted to optimize visualization of the thyroid parenchyma and lesion margins. When indicated, the bilateral cervical lymph nodes were also evaluated. Examinations were conducted using high‐frequency (4–13 MHz) linear‐array transducers (MyLab 90, Esaote; iU22, Philips; Resona 7, Mindray).

The following standardized ultrasound features were evaluated at both baseline and final follow‐up, with definitions and classifications based on the ACR TIRADS [11] and the C‐TIRADS [12]:

• Size and Volume: Maximum diameters measured in three orthogonal planes; volume calculated using the ellipsoid formula (*V* = *π* × *A* × *B* × *C*/6).
• Composition: Defined as solid (no cystic content) or predominantly solid (≤ 50% cystic component).
• Echogenicity: Categorized as hypoechoic, isoechoic, mixed, or hyperechoic.
• Orientation: Classified as horizontal or vertical.
• Margins: Circumscribed or irregular.
• Calcification: Absent, coarse, microcalcifications, or mixed.
• Vascularity: Evaluated via Doppler imaging; blood flow intensity graded as absent, minimal, moderate, or rich; vascular distribution categorized as peripheral, central, mixed, or none (Figure 1).
• Elasticity: When available, shear wave elastography assessed stiffness, categorized as soft, intermediate, or hard.

All ultrasound images were retrospectively reviewed and analyzed in consensus by two board‐certified radiologists with more than 5 years of experience in thyroid imaging. In cases of disagreement, a third radiologist with over 10 years of thyroid ultrasound experience provided the final decision. Parameters were compared between baseline and final follow‐up to evaluate longitudinal changes.

Figure 1Nodule structure and blood flow evaluation criteria: (a) Solid nodule; (b) predominantly solid nodule; (c) no detectable blood flow; (d) sparse blood flow; (e) moderate blood flow; and (f) rich blood flow.

**Figure:**
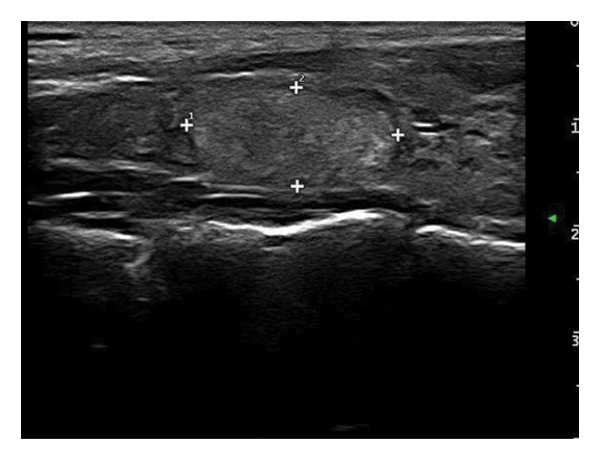
(a)

**Figure:**
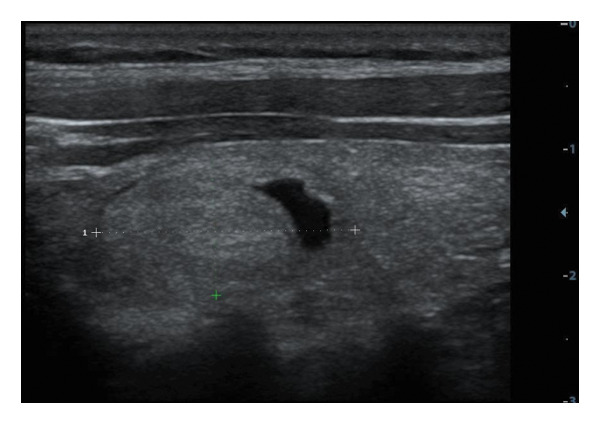
(b)

**Figure:**
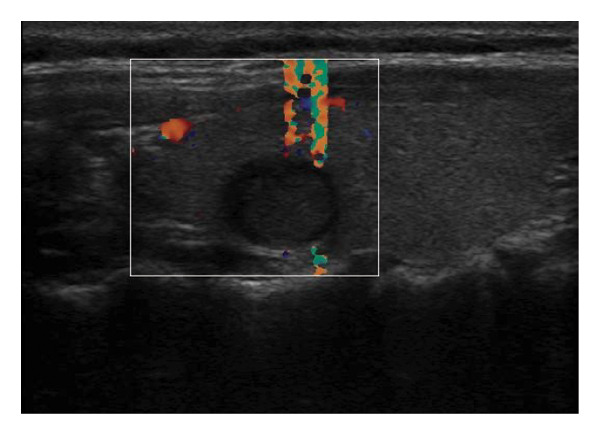
(c)

**Figure:**
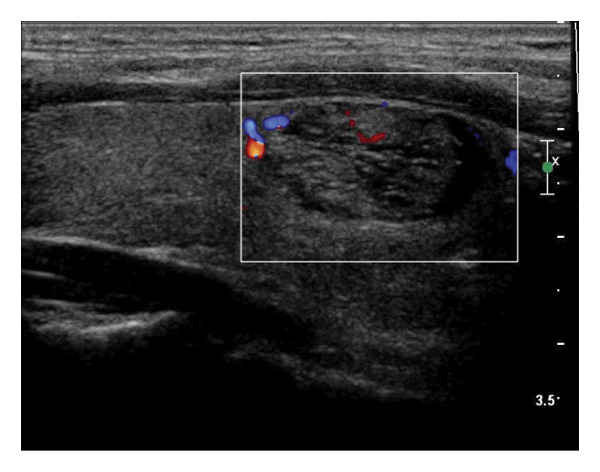
(d)

**Figure:**
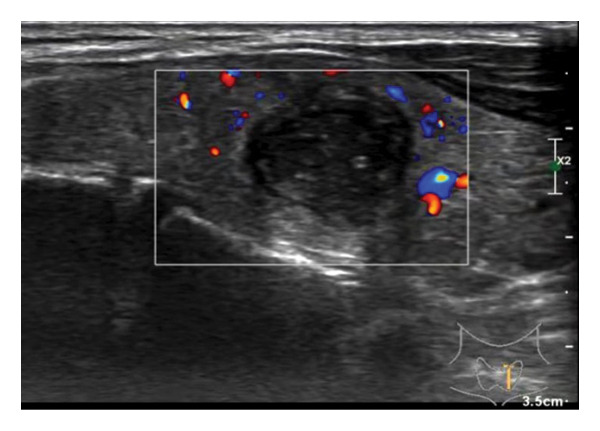
(e)

**Figure:**
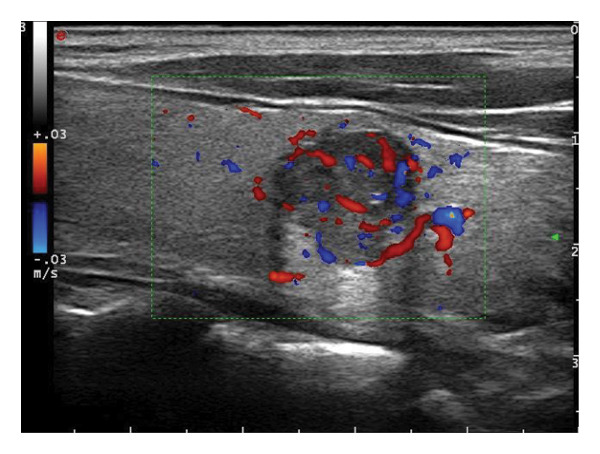
(f)

### 2.3. FNA and Molecular Testing

FNA was performed for selected nodules based on clinical indications and ultrasound features. Procedures were conducted under ultrasound guidance using a 22‐ or 25‐gauge needle via the long‐axis approach. Smears were prepared, fixed in ethanol, and stained for cytological evaluation. Cytology results were classified according to the Bethesda System. Molecular testing for *BRAF* and *TERT* promoter mutations was performed in selected cases to assess malignancy risk.

### 2.4. Statistical Analysis

Continuous variables were summarized using medians and interquartile ranges (IQR), and categorical variables were presented as counts and percentages. Intergroup comparisons of continuous data were performed using the Mann–Whitney *U* test due to the nonnormal distribution of nodule volume. Categorical variables were compared using Fisher’s exact test or chi‐square test, as appropriate, based on expected cell counts. To model longitudinal changes in nodule volume, a GAMM was constructed with time as a smooth term and patient‐specific random intercepts to account for within‐subject correlation. Log‐transformation of volume data was applied to reduce skewness and stabilize variance. Model fit was assessed using the effective degrees of freedom (EDF), adjusted *R* ^2^, and *F* statistics.

To identify representative regression trajectories, a two‐step unsupervised k‐means clustering procedure was applied. Two features were extracted from each individual’s longitudinal trajectory, including the slope of a subject‐level linear regression and the observed volume range, which were used as inputs for clustering. Outliers were defined as individual nodules whose longitudinal volume trajectories formed a unique single‐member cluster in the initial clustering step, indicating atypical behavior relative to the overall cohort. These outliers were excluded before the final clustering to ensure stable and interpretable cluster formation. The optimal number of clusters was determined by internal validity indices and a stability assessment. To visualize cluster‐specific dynamics, LOESS smoothing and cluster‐specific GAMM fits were performed. First derivatives of the GAMM‐fitted curves were computed using finite difference approximation to estimate the rate of volume change over time. To test whether the identified clusters represented distinct temporal patterns, an interaction GAMM including time × cluster terms was compared with a noninteraction model using a likelihood ratio test (LRT), and the corresponding ΔAIC and *p* values were reported. For internal validation, clusters were manually predicted based on interpretable clinical features such as nodule location and vascularity and then compared with the data‐driven clustering using a confusion matrix. Classification performance was evaluated by sensitivity, specificity, accuracy, and Cohen’s kappa coefficient.

All statistical analyses were performed in R (version 4.2.0) using the mgcv, nlme, ggplot2, and caret packages.

## 3. Results

### 3.1. Baseline Clinical and Nodule Characteristics

A total of 34 nodules from 32 patients were included in this study. The majority of patients were female (25/32, 78.1%), with a median age of 46 years (IQR: 39–53 years). The median follow‐up duration was 28 months (IQR: 47–120 months). A total of 15 (44.1%) nodules were identified in the left lobe, and 19 (55.9%) nodules were observed in the right lobe. The distribution of nodules revealed that 17 (50.0%) were located in the middle, 11 (32.4%) in the lower poles, and 6 (17.6%) in the upper poles (Table 1).

**Table 1:** Patient demographics and nodule characteristics.

Characteristics (total number)	Subcharacteristics	*N* (%)/median (IQR)
Gender (*n* = 32)	Female	25 (78.13%)
	Male	7 (21.87%)

Age (years)		46 (39–53)

Follow‐up time (months)		28 (47–120)

Lobe (*n* = 34)	Left	15 (44.12%)
	Right	19 (55.88%)

Location (*n* = 34)	Upper	6 (17.65%)
	Middle	17 (50.00%)
	Lower	11 (32.35%)

FNA (*n* = 10)	Benign	8 (80.00%)
	Malignant	0
	Undetermined	2 (20.00%)

BRAF (*n* = 7)	BRAF‐WT	7 (100%)
	BRAF‐Mut	0

TERT (*n* = 6)	TERT‐WT	6 (100%)
	TERT‐Mut	0

Maximum diameter (mm)	Initial	16.50 (10.55–22.23)
	Final	8.30 (6.33–11.78)

Volume (mm^3^)	Initial	869.17 (288.24–2548.56)
	Final	85.58 (68.31–95.32)

FNA was performed on ten nodules, yielding benign cytology in eight cases and indeterminate findings in two cases. No malignancy was detected at the initial evaluation. *BRAF* and *TERT* promoter mutation testing was performed on a limited subset of cases, all of which were negative for both mutations.

### 3.2. Longitudinal Changes in Ultrasonographic Features

The detailed comparison of ultrasonographic characteristics between the initial visit and the final follow‐up is presented in Table 2. At baseline, the median maximum diameter and volume of the nodules were 16.50 mm (IQR: 10.55–22.23 mm) and 869.17 mm^3^ (IQR: 288.24–2548.56 mm^3^), respectively. By the final follow‐up, these values had decreased to 8.30 mm (IQR: 6.33–11.78 mm) and 119.00 mm^3^ (IQR: 42.13–326.17 mm^3^), respectively. The overall median volume reduction rate was 85.58% (IQR: 68.31%–95.32%).

**Table 2:** Comparison of ultrasound features before and after follow‐up.

Ultrasound features	Subultrasound features	Initial visit	Final follow‐up
Composition	Solid	22 (64.7%)	30 (88.2%)
	Predominately solid	12 (35.3%)	4 (13.3%)

Echogenicity	Hypoechoic	12 (35.3%)	27 (79.4%)
	Isoechoic	9 (26.5%)	1 (2.9%)
	Mixed echo	12 (35.3%)	6 (17.6%)
	Hyperechoic	1 (2.9%)	0 (0%)

Orientation	Vertical	2 (5.9%)	3 (8.8%)
	Horizontal	32 (94.1%)	31 (91.2%)

Margin	Irregular margin/ill‐defined	1 (2.9%)	3 (8.8%)
	Circumscribed	33 (97.1%)	31 (91.2%)

Calcification	Macrocalcifications	2 (5.9%)	2 (5.9%)
	Microcalcifications	2 (5.9%)	4 (11.8%)
	Mixed calcification	0 (0%)	1 (2.9%)
	No calcification	30 (88.2%)	27 (79.4%)

Blood flow	No blood flow	11 (32.4%)	18 (52.9%)
	Minimal blood flow	11 (32.4%)	12 (38.2%)
	Moderate blood flow	8 (23.5%)	2 (5.9%)
	Rich blood flow	4 (11.8%)	2 (5.9%)

Vascular pattern	Mainly peripheral vascularity	17 (73.9%)	12 (75.0%)
	Mixed vascularity	6 (26.1%)	4 (25.0%)

Elastography	Soft	0 (0%)	1 (7.1%)
	Intermediate	2 (33.3%)	2 (14.3%)
	Hard	4 (66.7%)	11 (78.6%)

Ultrasound diagnosis	Benign	34 (100%)	19 (55.9%)
	Malignancy	0 (0%)	15 (44.1%)

During the subsequent follow‐up period, nodule composition demonstrated a tendency towards solidification. The proportion of solid nodules increased from 64.7% to 88.2%, while the proportion of predominantly solid nodules decreased from 35.3% to 13.3%.

A marked shift in echogenicity was observed. Initially, 35.3% of nodules were classified as hypoechoic; however, this proportion increased significantly to 79.4% at the final assessment. In contrast, isoechoic and mixed echo patterns exhibited a decline, from 26.5% to 35.3%–2.9% and 17.6%, respectively. Hyperechoic nodules manifested infrequently and exhibited complete resolution by the conclusion of the final follow‐up.

The orientation exhibited minimal alterations, with horizontal alignment maintaining its predominance (initial: 94.1%; final: 91.2%). Concurrently, margins were predominantly circumscribed throughout the study (initial: 97.1%; final: 91.2%), with a slight increase in irregular or ill‐defined margins at follow‐up (from 2.9% to 8.8%).

The calcification patterns exhibited a high degree of stability. The majority of nodules exhibited noncalcification (initial: 88.2%; final: 79.4%). Microcalcifications exhibited a modest rise (from 5.9% to 11.8%), while macrocalcifications remained stable at 5.9%. Mixed calcifications were observed in 2.9% of cases at the final follow‐up.

The results of the study indicated a tendency towards a reduction in vascularity, as evidenced by the blood flow characteristics. The proportion of nodules exhibiting no detectable blood flow increased from 32.4% to 52.9%, while those with moderate and rich blood flow decreased from 23.5% to 11.8%–5.9% and 5.9%, respectively. Peripheral vascular patterns predominated and remained consistent between baseline and follow‐up (initial: 73.9%; final: 75.0%).

On elastography assessment, the proportion of nodules categorized as “hard” increased notably from 66.7% to 78.6%, while the presence of “intermediate” stiffness decreased. At the time of the initial evaluation, none of the nodules were classified as “soft”; however, one case (7.1%) demonstrated softening at the subsequent evaluation.

Of particular significance were the alterations in sonographic interpretation that were also observed. Initially, all nodules were interpreted as benign. At the final follow‐up, 44.1% (15/34) of patients were reclassified as “suspected malignancy” based on imaging evolution, suggesting that despite overall volumetric shrinkage, echogenic and vascular changes may lead to reclassification during surveillance.

### 3.3. Temporal Dynamics of Nodule Absorption Patterns

To investigate the temporal absorption trends of thyroid nodules, we first constructed a GAMM using all the included nodules. As demonstrated in Figure 2(a), the fitted curve exhibited a discernible overall downward trend in nodule volume over time, indicating a global tendency toward shrinkage under surveillance. To better delineate internodule heterogeneity, the study excluded a single extreme outlier (at first clustering) and implemented unsupervised clustering based on individual volume trajectories. The convex hull plot (Figure 2(b)) delineated two discrete kinetic patterns. LOESS smoothing was performed to flexibly model intra‐cluster variation in volume change (Figure 2(c)). This analysis revealed a marked difference in absorption speed between the two clusters, representing relatively fast and slow absorptive behaviors. Subsequently, we implemented cluster‐specific GAMM to analyze the nodule volume data (Figure 2(d)). The fitted curves demonstrated a more rapid and substantial volume reduction rate in Cluster 1 compared to Cluster 2, thereby validating the kinetic differences suggested by the clustering methodology. The estimated absorption rate curves (Figure 2(e)) indicated that nodules in Cluster 1 achieved an absorption of 59.1% by 36 months, whereas Cluster 2 plateaued around 41.4% by 96 months. To quantify the speed of nodule shrinkage, the first derivative of the GAMM‐fitted curves was derived (Figure 2(f)). This analysis demonstrated that Cluster 1 had a substantially higher volume reduction rate, particularly during the first 12–48 months.

Figure 2Temporal shrinkage patterns of thyroid nodules under active surveillance: (a) GAMM fit of all nodules showing the overall volume reduction trend. (b) Convex hull plot showing two clusters after outlier exclusion and trajectory‐based clustering. (c) LOESS‐smoothed curves of volume over time for each cluster. (d) Cluster‐specific GAMM fits to original nodule volume data (non–log‐transformed). (e) Estimated absorption rate (%) over time by cluster. (f) First derivative of fitted GAMM curves, reflecting absorption velocity.

**Figure:**
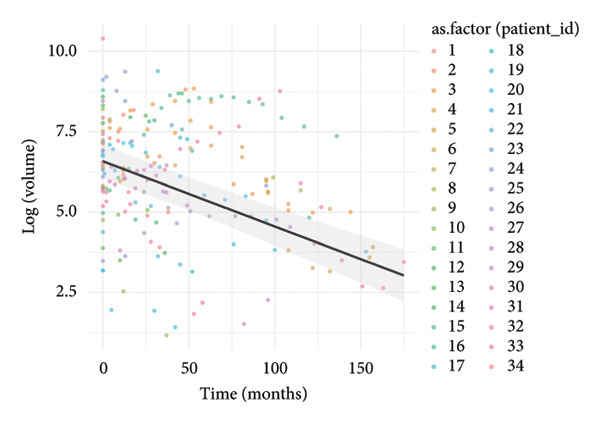
(a)

**Figure:**
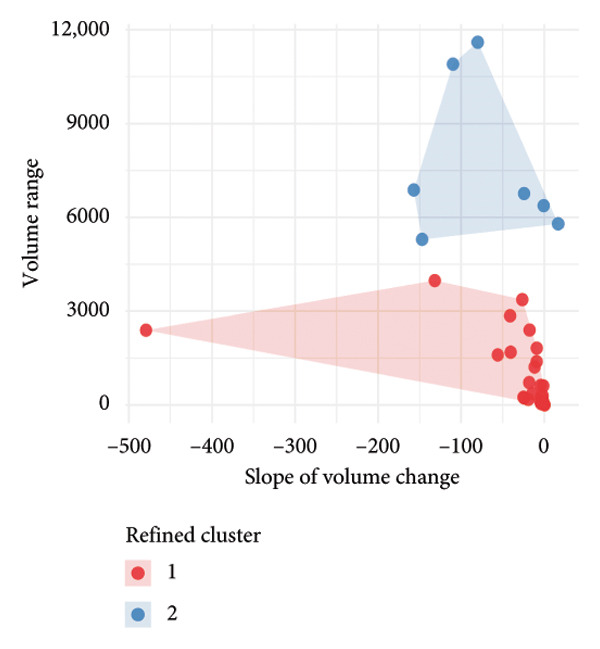
(b)

**Figure:**
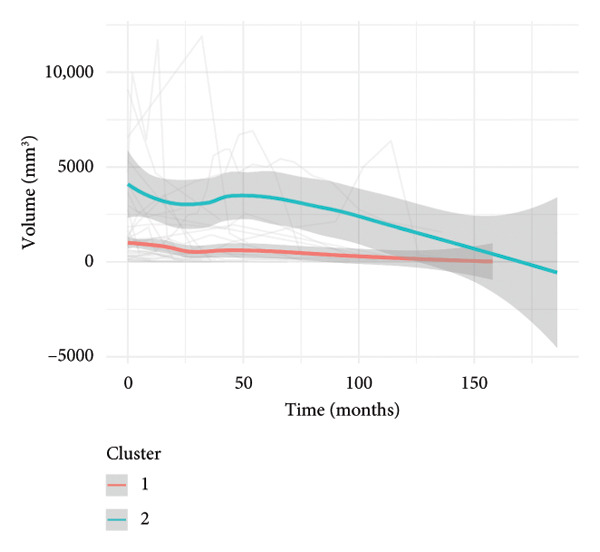
(c)

**Figure:**
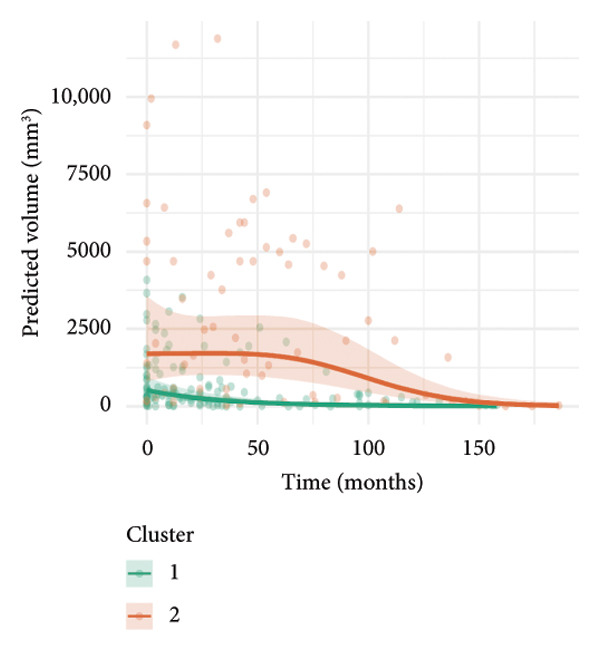
(d)

**Figure:**
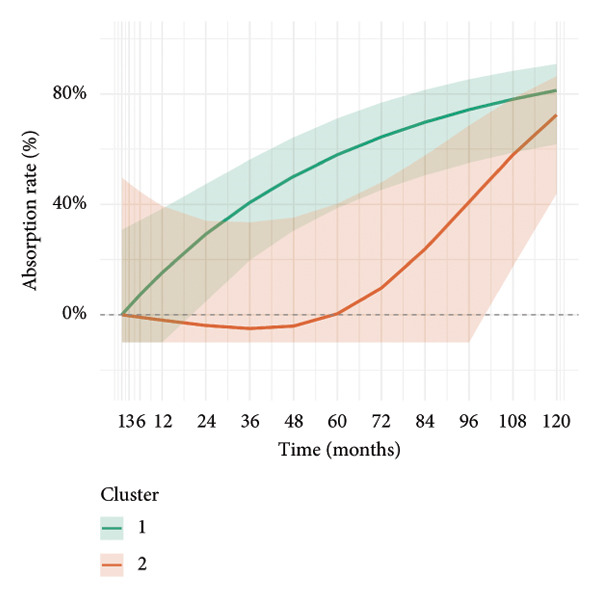
(e)

**Figure:**
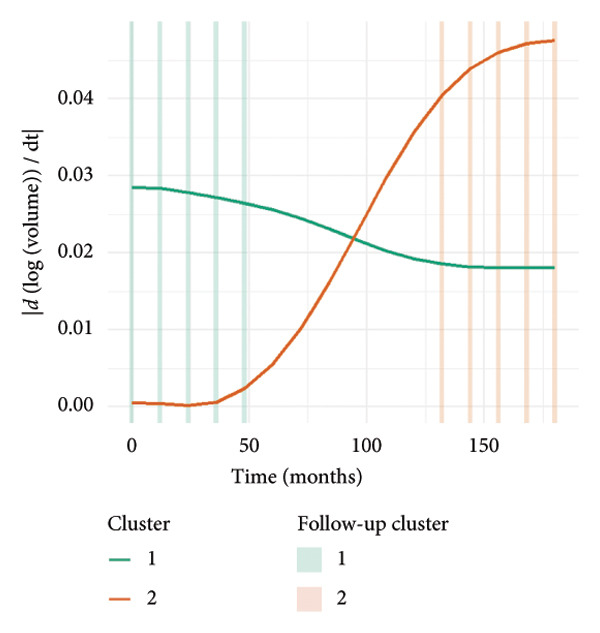
(f)

To assess whether the two clusters exhibited significantly distinct longitudinal behaviors in volume reduction, a GAMM incorporating an interaction between time and cluster was fitted. The interaction model demonstrated a statistically significant difference in volume trends between clusters (refined_cluster × time_point, *p* < 0.001). Specifically, cluster 1 exhibited an approximately linear decline in log‐transformed volume (EDF = 1.000, *F* = 63.67), whereas cluster 2 showed a more complex nonlinear pattern (EDF = 2.782, *F* = 12.17). Comparison with a noninteraction model confirmed the improved fit of the interaction model (ΔAIC = −16.64; likelihood ratio = 22.64, *p* < 0.0001). These findings indicate that the two clusters represent inherently different nodule absorption patterns over time, rather than being segments of the same trajectory.

### 3.4. Comparative Analysis of Baseline and Longitudinal Ultrasound Features Between Absorption Clusters

At baseline, nodules in Cluster 2 demonstrated a lower median volume compared to Cluster 1 (611.4 vs. 4690.9 mm^3^), with a borderline significant difference (*p* = 0.067). Cluster 2 was more frequently located in the lower pole of the thyroid (71.4% vs. 23.1%, *p* = 0.034), and a significantly higher proportion of these nodules exhibited absent vascular flow on Doppler imaging (85.7% vs. 19.2%, *p* = 0.015). No statistically significant differences were observed between clusters in echogenicity, composition, margin, calcification, or vascular pattern at either baseline or final follow‐up (all *p* > 0.05) (Table 3).

**Table 3:** Comparison of ultrasound features between Cluster 1 and Cluster 2 at baseline and final follow‐up.

Ultrasound features		Initial visit: nodule number (rate)		*p* value	Final follow‐up: nodule number (rate)		*p* value
		Cluster 1 (*n* = 26)	Cluster 2 (*n* = 7)		Cluster 1 (*n* = 26)	Cluster 2 (*n* = 7)	
Volume		4690.9 (786.7–5953.8)	611.4 (636.2–1495.8)	0.067	119.8 (39.2–287.3)	149.5 (67.7–1289.1)	0.33

Lobe	Left	10 (38.5%)	5 (71.4%)	0.203			
	Right	16 (61.5%)	2 (28.6%)				

Location	Upper	4 (15.4%)	1 (11.3%)	0.034			
	Middle	16 (61.5%)	1 (11.3%)				
	Lower	6 (23.1%)	5 (71.4%)				

Composition	Solid	18 (69.2%)	4 (57.1%)	0.661	24 (92.3%)	5 (71.4%)	0.19
	Predominately solid	8 (30.8%)	3 (42.9%)		2 (7.7%)	2 (28.6%)	

Echogenicity	Hypoechoic	10 (38.5%)	1 (11.29%)	0.601	21 (80.8%)	5 (71.4%)	0.676
	Isoechoic	6 (23.1%)	3 (42.86%)		1 (3.9%)	0 (0%)	
	Mixed echo	9 (34.6%)	3 (42.86%)		4 (15.4%)	2 (28.6%)	
	Hyperechoic	1 (3.9%)	0 (0%)		0 (0%)	0 (0%)	

Orientation	Vertical	2 (7.7%)	0 (0%)	1	3 (11.5%)	0 (0%)	1
	Horizontal	24 (92.3%)	7 (100%)		23 (88.5%)	7 (100%)	

Margin	Irregular margin/ill‐defined	1 (3.8%)	0 (0%)	1	3 (11.5%)	0 (0%)	1
	Circumscribed	25 (96.2%)	7 (100%)		23 (88.5%)	7 (100%)	

Calcification	Macrocalcifications	1 (3.9%)	1 (11.29%)	0.635	2 (7.7%)	0 (0%)	0.307
	Microcalcifications	2 (7.7%)	0 (0%)		3 (11.5%)	1 (11.3%)	
	Mixed calcification	0 (0%)	0 (0%)		0 (0%)	1 (11.3%)	
	No calcification	23 (88.5%)	6 (85.71%)		21 (80.8%)	5 (71.4%)	

Blood flow	No blood flow	5 (19.2%)	6 (85.71%)	0.015	12 (46.2%)	6 (85.7%)	0.411
	Minimal blood flow	10 (38.5%)	1 (11.29%)		10 (38.5%)	1 (11.3%)	
	Moderate blood flow	8 (30.8%)	0 (0%)		2 (7.7%)	0 (0%)	
	Rich blood flow	3 (11.5%)	0 (0%)		2 (7.7%)	0 (0%)	

Vascular pattern	Mainly peripheral vascularity	16 (61.5%)	1 (11.29%)	1	11 (42.3%)	1 (11.3%)	0.375
	Mixed vascularity	5 (19.2%)	0 (0%)		3 (11.5%)	0 (0%)	

### 3.5. Validation of Manually Predicted Clusters Based on Clinical Features

To assess whether clinical features could approximate the absorption pattern–based clusters, nodules were manually reassigned to predicted clusters using two features: location at the lower pole and absence of vascular flow. As shown in Figure 3(a), the GAMM‐fitted curves for the predicted clusters largely mirrored the actual clusters in both shape and absorption rate over time. The confusion matrix comparing these predicted clusters against the original clustering revealed an overall accuracy of 81.8% (*κ* = 0.55), indicating moderate agreement (Figure 3(b)). Sensitivity and specificity for identifying Cluster 1 were 80.8% and 85.7%, respectively. The predicted clusters were then fitted using the same GAMM approach. This consistency supports the feasibility of applying interpretable clinical features to infer underlying absorption trajectories.

Figure 3Comparison of actual and predicted cluster‐based GAMM curves: (a) GAMM‐based predicted volume trajectories over time for actual (Clusters 1 and 2) and clinically predicted clusters (based on location = lower pole and absence of blood flow). The solid lines indicate mean volume curves; shaded areas represent 95% confidence intervals. Close alignment between actual and predicted curves supports the biological plausibility of ultrasound‐based cluster classification. (b) Confusion matrix comparing these predicted clusters against the original clustering.

**Figure:**
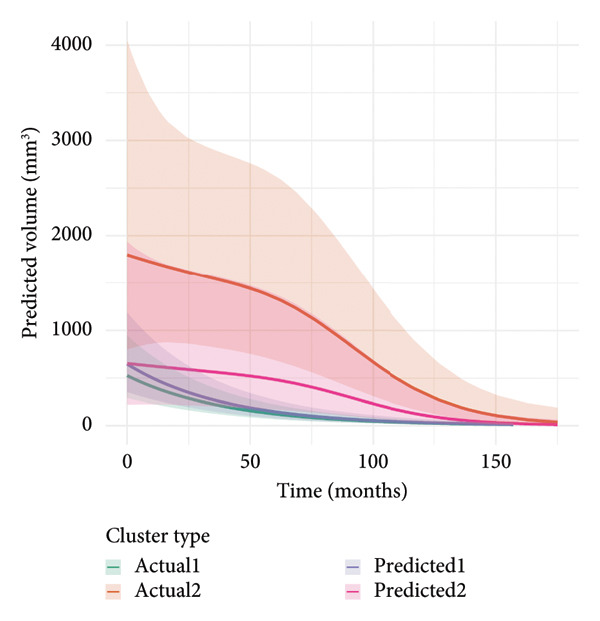
(a)

**Figure:**
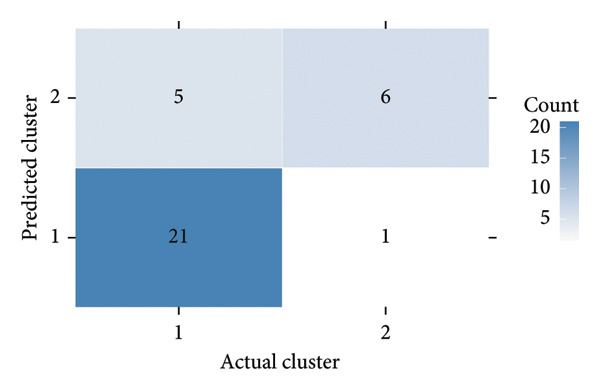
(b)

## 4. Discussion

This study systematically characterized the long‐term evolution of solid and predominantly solid thyroid nodules exhibiting measurable volume reduction or internal remodeling during ultrasound surveillance. Contrary to previous studies, which often included nodules with cystic or mixed compositions, our cohort was limited to solid‐dominant nodules on baseline imaging, a frequently overlooked subset in the existing literature. While these nodules are generally considered structurally stable, our findings suggest that they may gradually involute over time, accompanied by evolving ultrasonographic features. Unsupervised clustering of longitudinal volume trajectories revealed two distinct absorption patterns: a fast‐shrinking group (Cluster 1, Figure 4) and a slower, plateau‐like group (Cluster 2, Figure 5). These results imply that there is intrinsic heterogeneity in the regression behavior of sonographically benign nodules and offer new insights into the natural history of solid thyroid nodules.

Figure 4Progressive sonographic evolution of a solid thyroid nodule exhibiting a rapid absorption pattern a 64‐year‐old male patient was followed for 6 years: (a) At the initial follow‐up, the predominantly solid nodule measured 19 × 11 mm and appeared isoechoic. (b) Two years later, the size remained similar (19 × 9 mm), but the nodule began to exhibit mild hypoechogenicity. (c) At year 3, the nodule decreased in size to 12 × 8 mm and became distinctly hypoechoic. (d) By year 4, the nodule further shrank to 5 × 4 mm, with persistently marked hypoechogenicity. (e) At year 5, the nodule measured 5 × 3 mm, maintaining hypoechoic features. (f) After 6 years, the nodule had reduced to 4 × 3 mm, with sustained hypoechoic characteristics.

**Figure:**
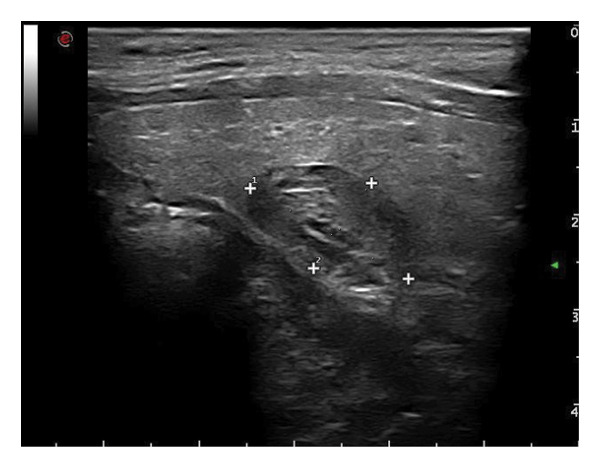
(a)

**Figure:**
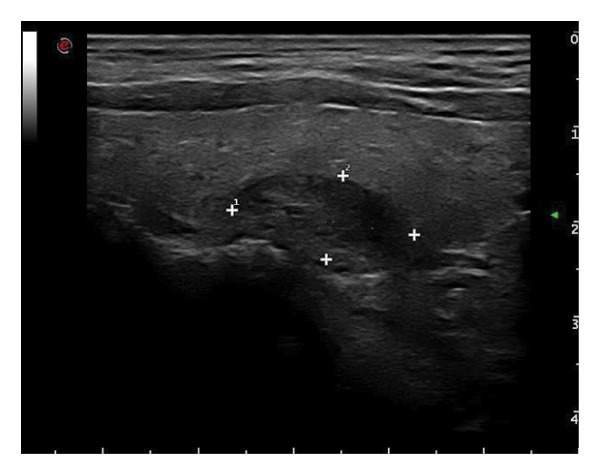
(b)

**Figure:**
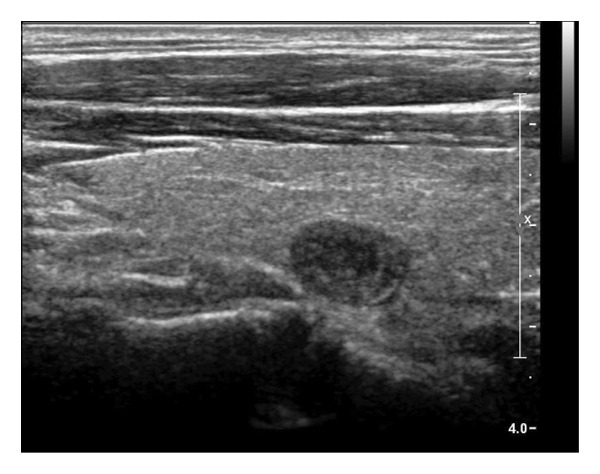
(c)

**Figure:**
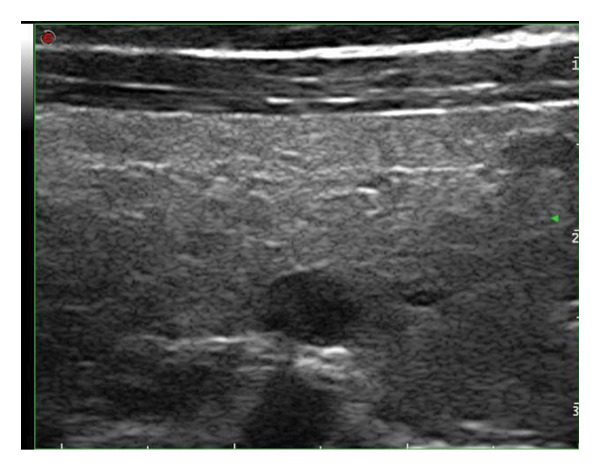
(d)

**Figure:**
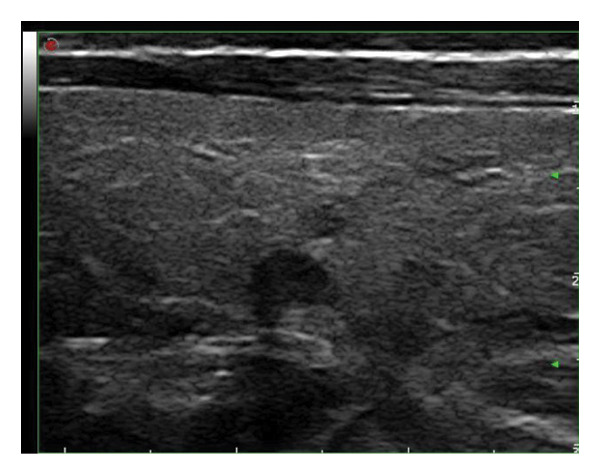
(e)

**Figure:**
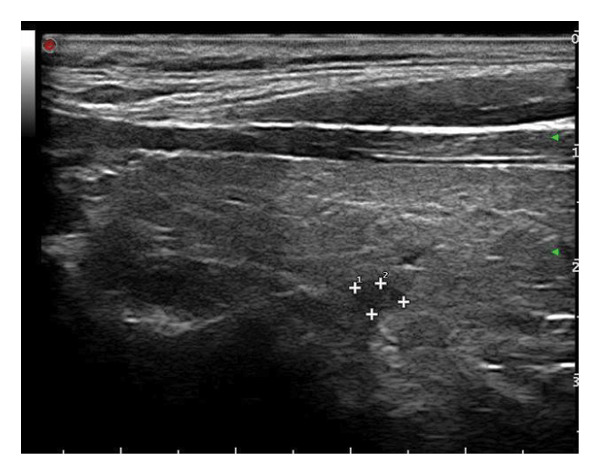
(f)

Figure 5Longitudinal ultrasound evolution of a predominantly solid thyroid nodule exhibiting a slow absorption pattern a 56‐year‐old male patient was followed for 13 years: (a) At initial follow‐up, the nodule measured 7 × 7 mm. (b) After 3 years, the nodule enlarged to 13 × 9 mm. (c) At 7 years, it increased further to 18 × 15 mm. (d) At 9 years, the size peaked at 28.2 × 20.8 mm. (e) By year 12, the nodule had markedly reduced in size to 3.5 × 5.2 mm, accompanied by the development of marked hypoechogenicity and a vertical orientation, raising sonographic suspicion. (f) At the 13‐year follow‐up, the nodule further decreased to 2.5 × 4.5 mm, while maintaining a hypoechoic appearance and vertical orientation.

**Figure:**
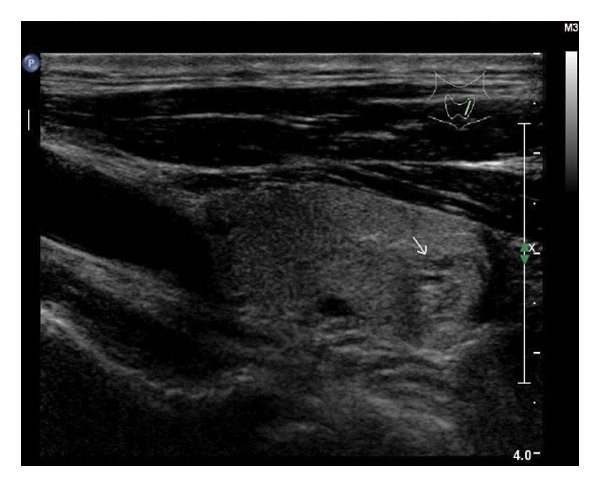
(a)

**Figure:**
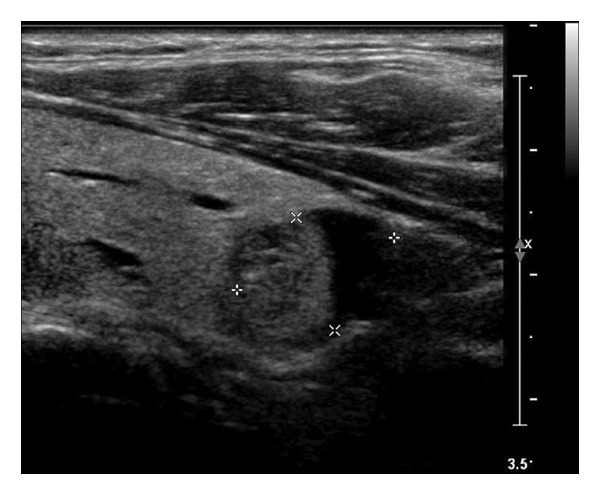
(b)

**Figure:**
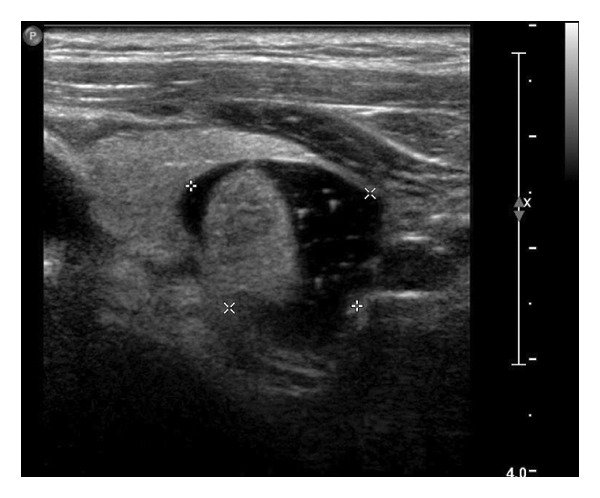
(c)

**Figure:**
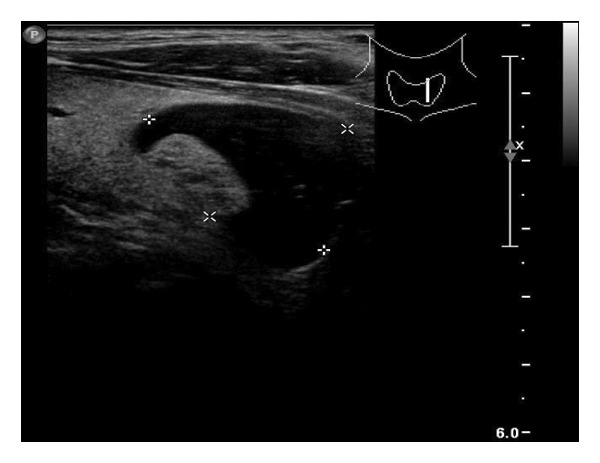
(d)

**Figure:**
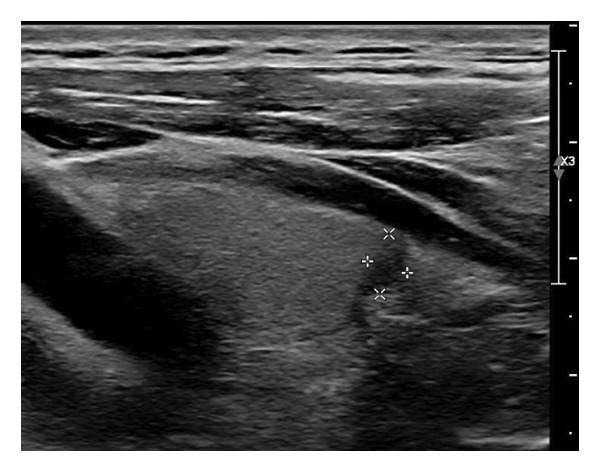
(e)

**Figure:**
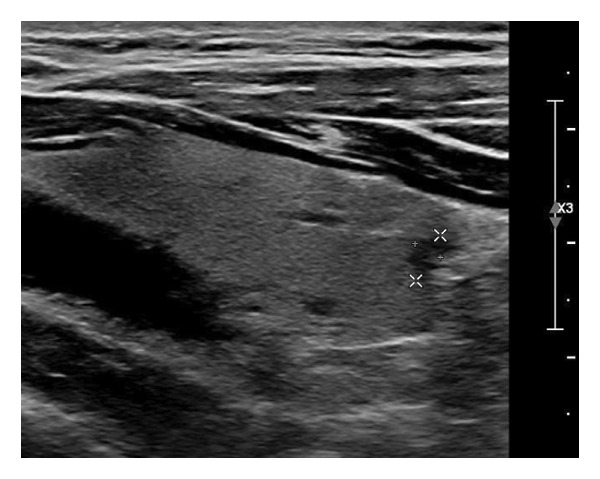
(f)

While existing literature has described shrinkage in benign nodules, these studies typically assessed heterogeneous nodule types, including cystic or mixed components. Durante et al. [16] reported that approximately 18% of cytologically benign nodules decreased in size over a 5‐year period but did not differentiate by nodule composition. Our study builds upon and extends this knowledge by demonstrating that even initially solid nodules can exhibit measurable regression, challenging the conventional notion that such nodules are unlikely to resolve.

In addition to volume reduction, we found that nodules frequently evolved sonographically during follow‐up. Increased hypoechogenicity, development of internal calcifications, and enhanced stiffness on elastography were observed in a substantial proportion of shrinking nodules. These features are typically associated with malignancy in static imaging but may also reflect benign degenerative changes such as fibrosis, hemorrhagic infarction, or necrosis [3]. Our findings align with previous observations by Lee et al. [17] and Ren et al. [6], who described “degenerating nodules” that mimicked malignancy despite being histologically benign. In our study, nearly half of the nodules were reclassified as “suspicious” based on evolving ultrasound features despite continuous size reduction and an absence of adverse outcomes. In this context, radiologists should evaluate serial examinations in relation to the overall volume trend and clinical stability instead of relying solely on static TIRADS features. Incorporating temporal changes into risk assessment may improve diagnostic specificity and help reduce unnecessary biopsies or interventions during follow‐up. Future large‐scale longitudinal studies are warranted to determine how temporal information can be incorporated into TIRADS frameworks to refine dynamic risk assessment during thyroid nodule surveillance.

We further observed that nodules with slower absorption trajectories were more frequently located in the lower thyroid pole and exhibited absent Doppler flow. These findings suggest that vascular and locational factors may influence the pace of nodule involution. Moreover, the use of simple clinical features to approximate kinetic clusters with over 80% accuracy suggests potential for future predictive modeling.

Our study has several limitations. The sample size was modest and derived from a single institution, which may limit generalizability. While most nodules were clinically stable without malignant transformation, pathological confirmation was limited to a small subset. The retrospective design also introduces possible selection and measurement bias, particularly with evolving ultrasound technology and operator variability over the surveillance period. Nonetheless, our application of modeling techniques such as GAMM and unsupervised clustering offers a robust framework for characterizing longitudinal volume change.

In summary, this study shows that, during long‐term ultrasound surveillance, a subset of solid and predominantly solid thyroid nodules undergoes volume reduction with internal absorption. Two distinct absorption trajectories were identified from longitudinal analyses.

**Nomenclature**
AIC: Akaike information criterion
ACR: American College of Radiology
ATA: American Thyroid Association
BRAF: B‐Raf proto‐oncogene
C‐TIRADS: Chinese Thyroid Imaging Reporting and Data System
EDF: Effective degrees of freedom
FNA: Fine‐needle aspiration
GAMM: Generalized additive mixed model
IQR: Interquartile range
LRT: Likelihood ratio test
LOESS: Locally estimated scatterplot smoothing
TERT: Telomerase reverse transcriptase
TIRADS: Thyroid Imaging Reporting and Data System

## Ethics Statement

This retrospective cohort study was approved by the institutional review board of the corresponding institution (Approval No. 2025122), with a waiver of informed consent due to its observational nature.

## Conflicts of Interest

The authors declare no conflicts of interest.

## Author Contributions

Yan Hu: writing–review and editing, writing–original draft, visualization, validation, supervision, software, resources, project administration, methodology, investigation, formal analysis, data curation, and conceptualization. Wei Zhou: software, conceptualization, writing–review and editing, visualization, validation, supervision, resources, project administration, methodology, and investigation. Shangyan Xu: methodology, formal analysis, and writing–review and editing. Guiping Zhang: investigation, writing–review and editing, resources, formal analysis, and data curation. Xiaoling Ma: methodology, validation, resources, and writing–review and editing. Lu Zhang: visualization, validation, supervision, project administration, methodology, funding acquisition, writing–review and editing, resources, and investigation. Weiwei Zhan: visualization, validation, supervision, project administration, methodology, funding acquisition, writing–review and editing, Resources, and investigation. Yan Hu and Wei Zhou contributed equally to this study.

## Funding

This work was supported by the National Natural Science Foundation of China (82071923).

## Supporting Information

The supporting information includes the completed STROBE checklist for this observational study.

## Supporting information


**Supporting Information** Additional supporting information can be found online in the Supporting Information section.

## Data Availability

The data that support the findings of this study are available on request from the corresponding authors. The data are not publicly available due to privacy or ethical restrictions.
